# Basal Cell Carcinoma: 10 Years of Experience

**DOI:** 10.1155/2011/476362

**Published:** 2010-12-01

**Authors:** Emanuele Cigna, Mauro Tarallo, Michele Maruccia, Valentina Sorvillo, Alessia Pollastrini, Nicolò Scuderi

**Affiliations:** Department of Plastic and Reconstructive Surgery, Policlinico Umberto I, 00161 Rome, Italy

## Abstract

*Introduction*. Basal cell carcinoma (BCC) is a locally invasive malignant epidermal tumour. Incidence is increasing by 10% per year; incidence of metastases is minimal, but relapses are frequent (40%–50%). The complete excision of the BCC allows reduction of relapse. *Materials and Methods*. The study cohort consists of 1123 patients underwent surgery for basal cell carcinoma between 1999 and 2009. Patient and tumor characteristics recorded are: age; gender; localization (head and neck, trunk, and upper and lower extremities), tumor size, excisional margins adopted, and relapses. *Results*. The study considered a group of 1123 patients affected by basal cell carcinoma. Relapses occurred in 30 cases (2,67%), 27 out of 30 relapses occurred in noble areas, where peripheral margin was <3 mm. Incompletely excised basal cell carcinoma occurred in 21 patients (1,87%) and were treated with an additional excision. *Discussion*. Although guidelines indicate 3 mm peripheral margin of excision in BCC <2 cm, in our experience, a margin of less than 5 mm results in a high risk of incomplete excisions.

## 1. Introduction

Basal-cell carcinoma (BCC) is a slow-growing, locally invasive malignant epidermal skin tumour. The lesion infiltrates tissues in a three-dimensional fashion [1] through the irregular growth of subclinical finger-like outgrowths which remain contiguous with the main tumour mass [2, 3]. Metastasis is extremely rare [4, 5], and morbidity results from local tissue invasion and destruction, particularly on the face, head, and neck. 

Incidence of basal-cell carcinoma alone is increasing by 10% per year worldwide, suggesting that prevalence of this tumor will soon equal that of all other cancers combined [6].

Although the incidence of metastases is minimal, relapses are, in the other hand, very frequent; indeed, an estimated 40%–50% of patients with a primary carcinoma will develop at least one or more further basal-cell carcinomas within 5 years [7]. According to a meta-analysis of 16,066 cases, recurrence rates for 5 mm, 4 mm, 3 mm, and 2 mm surgical margins were 0.39, 1.62, 2.56, and 3.96 percent, respectively [8]. Mohs technique recurrence rate is 4,5% in five years [9].

A wide range of different treatments has been described for the management of BCC [10]. Some techniques, as cryosurgery, curettage, RT, photodynamic therapy do not allow histological confirmation of tumour clearance. Differently surgical excision with either intraoperative or postoperative histological assessment of the surgical margins is widely used to treat BCC and is generally considered to have the lowest overall failure rate in BCC treatment [11].

The complete excision of the BCC allows disease control and reduction of relapse considered and is an appropriate surgical treatment which must focalize on the peripheral margin.

## 2. Materials and Methods

The study cohort consists of 1123 patients who underwent surgery for basal-cell carcinoma between 1999 and 2009. All the cases were treated in Department of Plastic and Reconstructive surgery of Policlinico Umberto I. Including criteria are: positivity to BCC at histological analysis and follown up period longer than 1 year. Patients with inadequate follown up, defined as <1 year or <2 visits per year were excluded from the analysis. 

The excision of the neoplasm was performed by the same plastic surgeon as well as histological evaluation was performed by the same pathologist during the study period. Specimens were histologically evaluated to determine the predominant histologic subtype (superficial, nodular, infiltrative BCC or mixed pattern consisting of both nodular and infiltrative BCC subtypes).

Patients' followup was made by a plastic surgeon or by a dermatologist related to Dermatology, Plastic, and Reconstructive Surgery Department. 

The following patient and tumor characteristics were recorded: age, gender, anatomic location (head and neck, trunk, and upper and lower extremities), tumor size, excisional margins adopted, and relapses.

## 3. Results

The study considered a group of 1123 patients affected by basal-cell carcinoma. Average age was 64,5 years old. A relevant difference came out between two genders. 764 Males (68%) were affected in comparison with only 359 females (32%). Concerning areas affected, first was cervicofacial area with a prevalence of 652 cases (58%), second trunk 256 cases (23,5%), third lower limbs 97 cases (8,9%), fourth upper limbs 71 (6%), and other areas 47 cases (3,6%).

Average diameter of lesions was 12.2 mm; the biggest lesion measured 5.3 cm, the smallest 0.2 cm. Margins taken were 3 to 5 mm on cervico-facial area, 2-3 mm on noble areas as lips, ears, and eyelid and 5 to 10 mm on other areas. An additional excision of 3 mm from the scar's margin was performed in incompletely excised neoformations. 

Relapses occurred in 30 cases (2.67%); 27 (90%) out of the 30 relapses were located in noble areas in which the initial peripheral margin of excision was <3 mm. Two further relapses were located at the neck and one at the trunk. 

A relation between relapses and histological subtype was evident. In effect, morphoeic-infiltrative subtype accounted for 63% (18 cases) of relapsed forms, micronodular subtype, superficial subtype, while nodular subtype accounted for 31.5% (9 cases), 7% (2 cases), and 3,5% (1 case), respectively. Incompletely excised basal cell carcinoma occurred in 21 patients (1.87%) and were treated with an additional excision. No metastases were documented.

## 4. Discussion

Optimal management of basal-cell carcinoma is increasingly relevant in an aging population in which BCC is the fifth most costly cancer [12].

Because of great increase in incidence of this cancer, a detailed analysis on what are risk factors and how best to manage BCC was carried out by many authors [7, 13–15].

The main risk factor is sun exposure, because UVB radiation causes direct damage to DNA and RNA by inducing covalent bond formation between adjacent pyrimidines, leading to generation of mutagenic photoproducts such as cyclopyrimidine dimers (TT) and pyrimidine-pyrimidine (6–4) adducts [14]. UVA is less mutagenic than is UVB, and causes indirect DNA damage via a photo-oxidative stress-mediated mechanism, resulting in formation of reactive oxygen species, which interact with lipids, proteins, and DNA to generate intermediates that combine with DNA to form adducts [16]. Several complex DNA repair systems are needed to prevent the harmful effects of these premutagenic adducts [17].

As a matter of fact, a mutation in the genes coding for repair proteins are involved in numerous syndromes characterized by the proliferation of basal-cell carcinoma. An example of these syndromes are Gorling-Goltz one, produced due to a mutation in patched gene (PTCH), located in the 9q22.3 chromosome, with loss of heterozygosity. This gene is involved in a great variety of processes such as the embryogenesis, homeostasis maintenance in the old tissues, tissue repairing during the persistent chronic inflammation and carcinogenesis [18, 19].

Although the understanding of BCC pathogenesis is greatly improved, incidence of the disease is steadily increasing, and the surgeon's task is to assess the best BCC management.

Factors indicating poor prognosis in BCC are a size >5 cm (giant), morphemic clinical subtype, tumour depth, infiltrative and micronodular histological subtype, and recurrent lesions. In addition, systemic alteration can be connected to a worse prognosis such as immunosuppression and distant metastasis. Another feature of bad prognosis recurrent lesions hence is incomplete excision should be considered a poor prognostic factor, since patients with an inadequately excised lesion present a higher risk of local recurrence [20–23]. An assessment of last two points leads to the assumption that a complete excisional treatment improves the prognosis of the patient by limiting relapses.

Although numerous different treatments have been described for the management of BCC, surgical excision is a highly effective treatment for primary lesions [24, 25]. According to our data, recurrence rate is <2% after a complete excision. In order to perform the best surgical treatment, the size of the peripheral and deep surgical margins should correlate with the likelihood that subclinical tumour extensions exist. According Griffiths et al. [26], deep margin clearance is in the range between 0,1 and 9.9 mm. Generally, as this will depend upon the local anatomy, excision through subcutaneous fat is advisable.

According to the guidelines [27], excision of small (<20 mm) well-defined lesions with a 3 mm peripheral surgical margin will clear the tumour in 85% of cases. A 4-5 mm peripheral margin will increase the peripheral clearance rate to approximately 95%, indicating that approximately 5% of small, well-defined BCCs extend over 4 mm beyond their apparent clinical margins.

Although guidelines indicate as 3 mm peripheral margin of excision in BCC <2 cm, in our experience, a margin of less than 5 mm results in a high risk of incomplete excisions. Almost all the recurrences in our series (90%) occurred in noble areas, in which the peripheral margin was minimal. This indicates clearly that a minimum margin of excision (<3 mm) increases considerably the risk of relapse, even if the histology shows complete excision of lesion.

 Similar data are reported by Griffiths et al. who stated that [26] only 65% of BCC excisions had peripheral clearance margins in the range of 0.1–4.9 mm. According to Madan et al. [7], a 4-5 mm surgical margin ensures peripheral clearance in roughly 95% of well-defined small basal-cell carcinomas.

**Figure 1:**
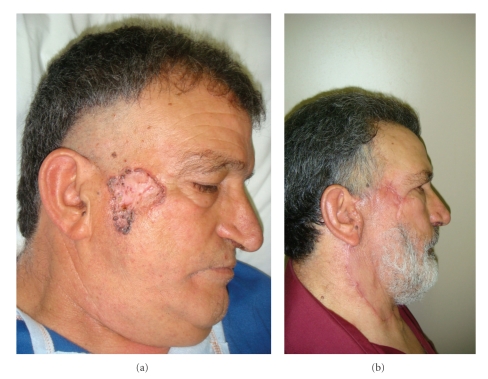
(a) and (b) Pre- and postintervention on zygomatic region.

**Figure 2:**
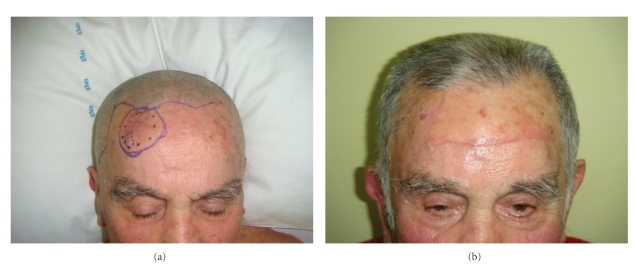
(a) and (b) Pre- and postoperative on forehead.

**Figure 3:**
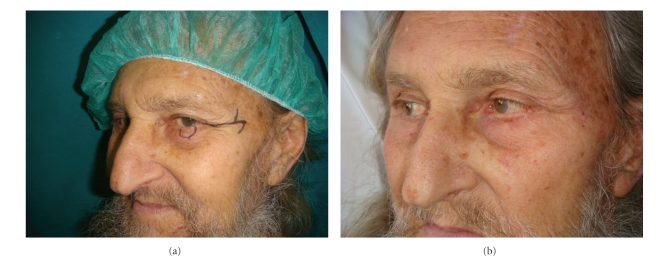
(a) and (b) Pre-post operative on lower eye-lid.

So no way a margin of 3 mm can be considered adequate in BCC excision. This minimum margin may be considered for some particular areas as nose, eyelid, and lips, for a better functional and aesthetic result (Figures 1(a), 1(b), 2(a), 2(b), 3(a), 3(b)).

In back- to legs- areas, a margin of excision ranging from 0.5 cm and 1 cm can be used, creating no significant changes in function and aesthetic and ensuring complete clearance.

According to Smeets et al. [9], the overall 5-year recurrence rate is 4,5%, using the Mohs surgery against a recurrence rate of 3.96% with a peripheral margin of 3 mm. These results highlights that although aesthetic results using Mohs technique are very good, from an oncological radicality standpoint Mohs technique is inferior to classical excision.

## 5. Conclusions

This paper focuses on how evaluation and standardization of excisional margins is a priority for a correct surgical activity. 

An excisional margin >4 mm should be ensured in facial area and a margin >6 mm in other body areas. Only on rare occasions, excision can be performed with a peripheral margin of 3 mm. Then, a differentiation on excisional margins based on the location of the lesion should be made to ensure the best result from an oncological, functional, and aesthetic standpoint.
